# Cancer cell death induced by novel small molecules degrading the TACC3 protein via the ubiquitin–proteasome pathway

**DOI:** 10.1038/cddis.2014.471

**Published:** 2014-11-06

**Authors:** N Ohoka, K Nagai, T Hattori, K Okuhira, N Shibata, N Cho, M Naito

**Affiliations:** 1 Division of Biochemistry and Molecular Biology, National Institute of Health Science, Kamiyoga, Setagaya-ku, Tokyo 158-8501, Japan; 2 Medicinal Chemistry Research Laboratories, Pharmaceutical Research Division, Takeda Pharmaceutical Co. Ltd., 26-1, Muraoka-Higashi 2-chome, Fujisawa, Kanagawa 251-0012, Japan

## Abstract

The selective degradation of target proteins with small molecules is a novel approach to the treatment of various diseases, including cancer. We have developed a protein knockdown system with a series of hybrid small compounds that induce the selective degradation of target proteins via the ubiquitin–proteasome pathway. In this study, we designed and synthesized novel small molecules called SNIPER(TACC3)s, which target the spindle regulatory protein transforming acidic coiled-coil-3 (TACC3). SNIPER(TACC3)s induce poly-ubiquitylation and proteasomal degradation of TACC3 and reduce the TACC3 protein level in cells. Mechanistic analysis indicated that the ubiquitin ligase APC/C^CDH1^ mediates the SNIPER(TACC3)-induced degradation of TACC3. Intriguingly, SNIPER(TACC3) selectively induced cell death in cancer cells expressing a larger amount of TACC3 protein than normal cells. These results suggest that protein knockdown of TACC3 by SNIPER(TACC3) is a potential strategy for treating cancers overexpressing the TACC3 protein.

Inhibitors of microtubule polymerization or depolymerization such as *Vinca* alkaloids and taxanes, respectively, are widely used as anti-cancer drugs. They arrest cancer cells, inducing mitotic catastrophe and cancer cell death. However, these drugs also affect microtubule function in non-dividing cells and have serious side effects, such as peripheral neuropathy, which limit their utility.^1^ Recently, inhibitors of spindle-regulatory proteins, such as mitotic kinases (Aurora kinases and Polo-like kinases) and a motor protein (Eg5/Ksp) have attracted considerable attention, but they have not been developed clinical use yet.^2, 3^

Transforming acidic coiled-coil-3 (TACC3) is another spindle-regulatory protein.^4, 5^ During mitosis, TACC3 localizes to the mitotic spindle and has a critical role in spindle assembly, chromosomal function and mitotic progression.^6, 7, 8, 9, 10, 11^ Studies using microarray and immunohistochemical analysis showed that TACC3 is overexpressed in many human cancers, including ovarian cancer, breast cancer, squamous cell carcinoma and lymphoma.^12, 13, 14^ Depletion of TACC3 results in chromosome alignment defects, multi-polar spindle formation, mitotic cell death and/or a postmitotic cell cycle arrest.^15, 16, 17, 18, 19, 20^ Additionally, conditional disruption of TACC3 has been shown to regress thymic lymphomas in p53-deficient mice without inducing any overt abnormalities in normal tissues.^21^ These findings suggest that TACC3 is a molecular target for anti-cancer drug discovery.

The development of a strategy for the selective degradation may be a useful approach to the discovery of novel drugs. Based on the ubiquitin–proteasome system (UPS), we have devised a protein knockdown system for inducing the selective degradation of target proteins by using specifically designed hybrid small compounds.^22, 23, 24, 25, 26, 27, 28, 29^ These compounds, which we have termed SNIPER (Specific and Non-genetic IAP-dependent Protein ERaser), are composed of two different ligands connected by a linker; one is a ligand for cellular inhibitor of apoptosis protein 1 (cIAP1) and the other a ligand for the target protein. Accordingly, SNIPER is expected to crosslink the ubiquitin–ligase cIAP1 and the target protein in the cells, thereby inducing ubiquitylation and, ultimately, proteasomal degradation of the target protein. To date, we have constructed SNIPERs that target cellular retinoic acid binding protein-II (CRABP-II) and nuclear receptors such as estrogen receptor *α* (ER*α*) for degradation.^22, 23, 24, 25, 26, 27, 28^ In this study, we designed and synthesized novel SNIPERs targeting TACC3, that is, SNIPER(TACC3)s, that induce proteasomal degradation of the TACC3 protein. We also show that cancer cells expressing a large amount of the TACC3 protein readily undergo cell death as the result of SNIPER(TACC3) treatment.

## Results

### Effect of SNIPER(TACC3) on TACC3 protein expression

**Figure 1:**
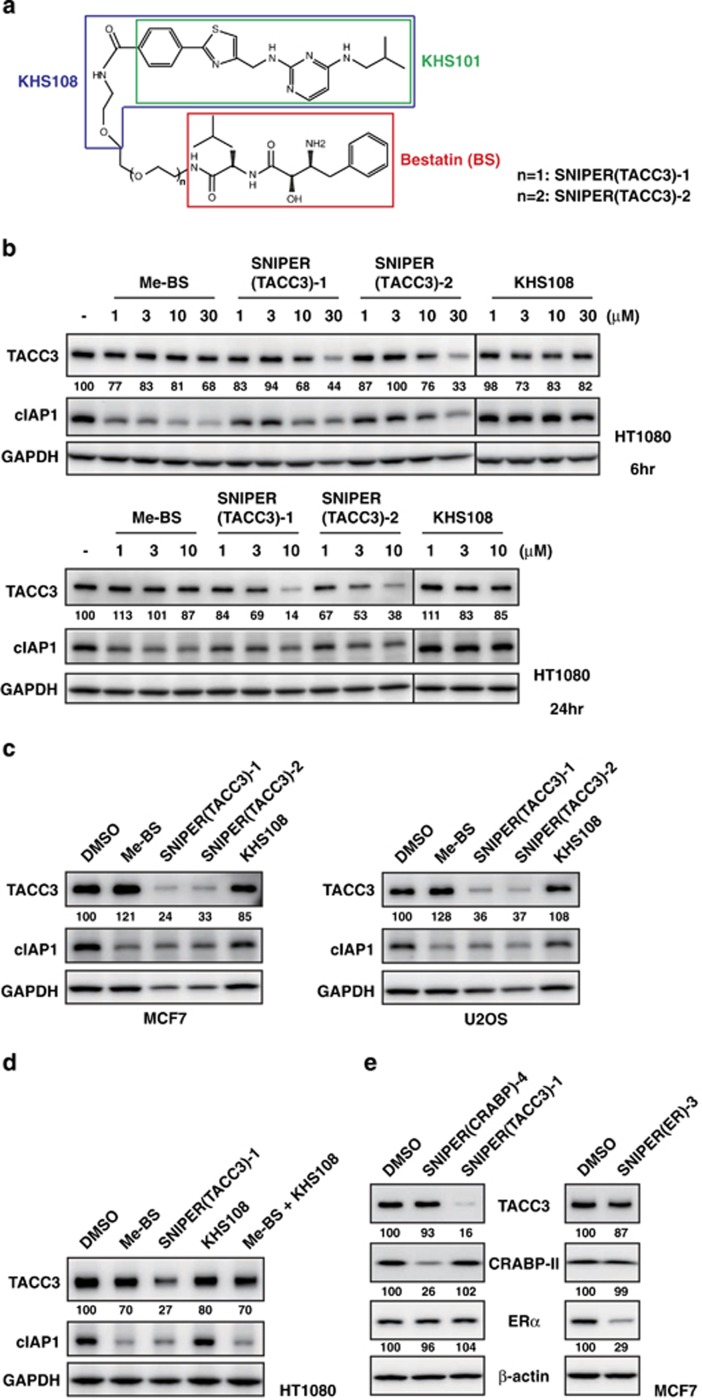
SNIPER(TACC3) decreases the TACC3 protein level. (**a**) Chemical structure of SNIPER(TACC3). (**b**) HT1080 cells were treated with the indicated concentration of Me-BS, SNIPER(TACC3)-1, SNIPER(TACC3)-2 or KHS108 for 6 h (upper panels) or 24 h (lower panels). (**c**) MCF7 and U2OS cells were treated with 30 *μ*M of Me-BS, SNIPER(TACC3)-1, SNIPER(TACC3)-2 or KHS108 for 6 h. (**d**) HT1080 cells were treated with 30 *μ*M of Me-BS, SNIPER(TACC3)-1, KHS108 or Me-BS plus KHS108 for 6 h. (**e**) MCF7 cells were treated with 10 *μ*M of SNIPER(TACC3)-1 or SNIPER(CRABP)-4 for 6 h (left panels), or cells precultured in the media containing estrogen-depleted serum were treated with 10 *μ*M of SNIPER(ER)-3 for 9 h (right panels). (**b**–**e**) Whole-cell lysates were analyzed using western blotting with the indicated antibodies. The numbers below the protein panels represent the protein level relative to control (dimethyl sulfoxide (DMSO)), which was normalized by glyceraldehyde 3-phosphate dehydrogenase (GAPDH) or *β*-actin

We designed and synthesized the SNIPER(TACC3) to target the TACC3 protein for degradation (Figure 1a). The synthesis and structural data on SNIPER(TACC3)-1 and -2 are presented in Materials and Methods section and Supplementary Information. When human fibrosarcoma HT1080 cells were treated with graded concentrations of SNIPER(TACC3)-1 or -2, the TACC3 level was significantly decreased by these compounds at 30 *μ*M for 6 h and at 10 *μ*M for 24 h (Figure 1b). However, methyl-bestatin (Me-BS) or KHS108 marginally reduced the TACC3 protein level under the same condition. SNIPER(TACC3)s also decreased the cIAP1 level, although the effect was less than Me-BS, suggesting that the SNIPER(TACC3)s simultaneously induces auto-ubiquitylation and proteasomal degradation of cIAP1, as observed with other SNIPERs.^22, 28^ Similar results were obtained in human breast adenocarcinoma MCF7 or human osteosarcoma U2OS cells when the cells were treated with 30 *μ*M of SNIPER(TACC3)s for 6 h (Figure 1c). Combination treatment with Me-BS and KHS108 did not decrease the TACC3 protein level, indicating that linking the two ligands into a single molecule is critically important for the reduction of the TACC3 protein (Figure 1d). Additionally, we examined the target specificity of the SNIPERs we have developed. SNIPER(TACC3), SNIPER(CRABP) and SNIPER(ER) reduced the level of respective target proteins without reducing the other proteins (Figure 1e), indicating the specificity of SNIPERs on the degradation of the target proteins.

### Ubiquitylation and proteasomal degradation of TACC3 protein by SNIPER(TACC3)

**Figure 2:**
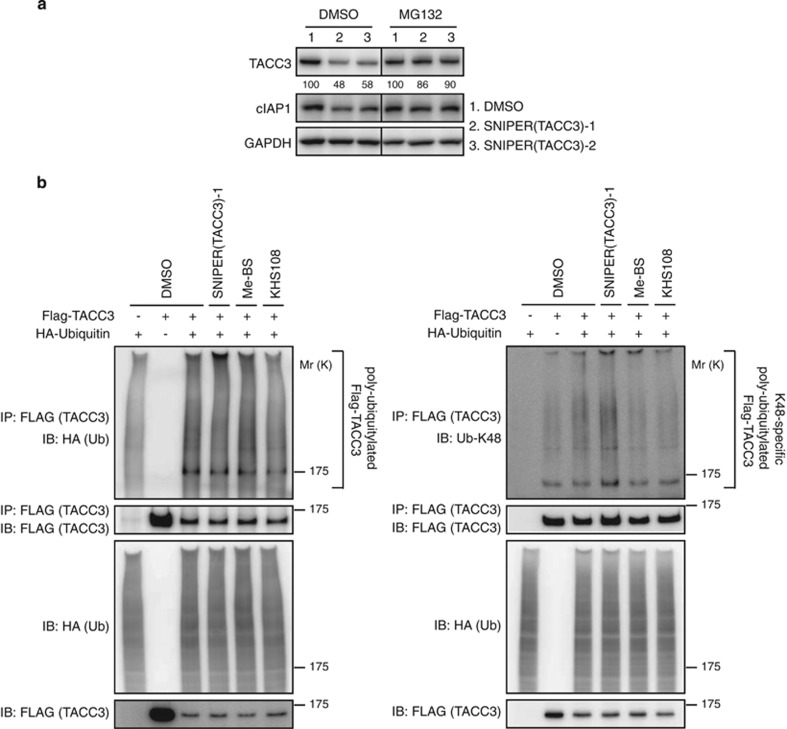
SNIPER(TACC3) induces ubiquitylation and proteasomal degradation of TACC3. (**a**) HT1080 cells were treated with 30 *μ*M of SNIPER(TACC3)-1 or -2 in the presence or absence of 25 *μ*M of MG132 for 4 h. Whole-cell lysates were analyzed using western blotting with the indicated antibodies. The numbers below the TACC3 panels represent the TACC3 level relative to the respective control normalized by glyceraldehyde 3-phosphate dehydrogenase (GAPDH). (**b**) HT1080 cells were co-transfected with the expression vector of Flag-TACC3 and/or HA-ubiquitin. After 40 h, cells were treated with the indicated compounds in the presence of 25 *μ*M of MG132 for 3 h. Cells were lysed, and Flag-TACC3 was immunoprecipitated with an anti-FLAG antibody. The ubiquitylated TACC3 was detected with an anti-HA antibody (left panels) or an antibody specific to K48-linked ubiquitin (right panels). DMSO, dimethyl sulfoxide; IB, immunoblot; IP, immunoprecipitation

To investigate whether the reduction of the TACC3 protein by SNIPER(TACC3) is mediated by proteasomal degradation, HT1080 cells were co-treated with SNIPER(TACC3) and the proteasome inhibitor MG132. As shown in Figure 2a, the decrease in the TACC3 protein by SNIPER(TACC3)s was abrogated by MG132, indicating that SNIPER(TACC3)s reduce TACC3 protein by a proteasome-dependent mechanism.

We next examined the effect of SNIPER(TACC3) on the ubiquitylation of TACC3. HT1080 cells were transiently co-transfected with the expression vectors of Flag-tagged TACC3 and HA-tagged ubiquitin and then treated with SNIPER(TACC3)-1 or control compounds in the presence of MG132. The cell lysates were heat-denatured to dissociate non-covalently bound proteins, then re-natured and immunoprecipitated with anti-FLAG (TACC3) antibody. The immunoprecipitates were subsequently analyzed by western blotting with an anti-HA (ubiquitin) to detect ubiquitylated TACC3 proteins (Figure 2b, left panels). SNIPER(TACC3)-1 treatment, but not Me-BS or KHS108, shifted the smear bands of the poly-ubiquitylated TACC3 protein to a more slowly migrating position (near the top of gel), indicating that SNIPER(TACC3) induces a higher level of poly-ubiquitylation of the TACC3 protein. We carried out a similar experiment with an antibody specific to K48-linked ubiquitin and observed a more conspicuous enhancement of K48-polyubiquitylated TACC3 protein by SNIPER(TACC3), but not by Me-BS nor KHS108 (Figure 2b, right panels). These results indicate that SNIPER(TACC3)s induce poly-ubiquitylation and proteasomal degradation of TACC3 proteins within cells.

### SNIPER(TACC3)-induced degradation of TACC3 protein requires APC/C^CDH1^

**Figure 3:**
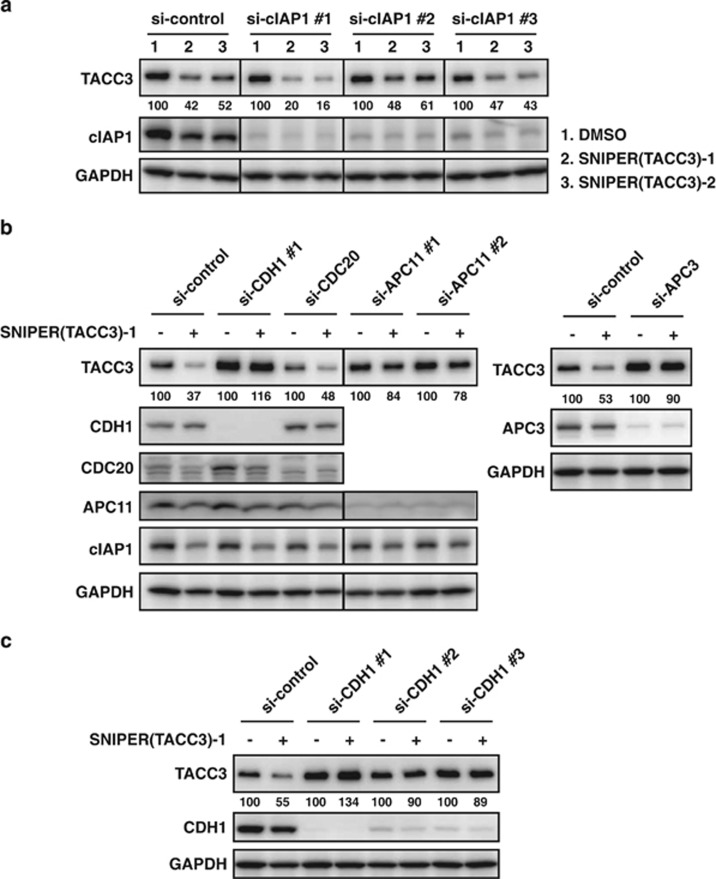
APC/C^CDH1^ mediates SNIPER-induced TACC3 reduction. (**a**–**c**) HT1080 cells were transfected with the indicated siRNA for 40 h and treated with 30 *μ*M of SNIPER(TACC3)-1 for 4 h. Whole-cell lysates were analyzed using western blotting with the indicated antibodies. The numbers below the TACC3 panels represent the TACC3 level relative to the respective control normalized by glyceraldehyde 3-phosphate dehydrogenase (GAPDH). DMSO, dimethyl sulfoxide

As SNIPER(TACC3) is designed to crosslink the ubiquitin ligase cIAP1 to the TACC3 protein, we reasoned that the TACC3 protein is degraded subsequent to cIAP1-mediated ubiquitylation. To examine whether cIAP1 is actually involved in the SNIPER(TACC3)-induced TACC3 degradation, we pretreated the cells with small interfering RNA (siRNA) against cIAP1 and measured the reduction of TACC3 protein by SNIPER(TACC3)s. We used three different siRNAs against cIAP1, and they all efficiently downregulated the cIAP1 protein level. Unexpectedly, however, the downregulation of the cIAP1 protein did not abrogate the reduction in the TACC3 protein by SNIPER(TACC3)s (Figure 3a). This result suggests that the TACC3 protein is ubiquitylated by a different ubiquitin ligase than cIAP1 in the SNIPER(TACC3)-treated cells.

As an inherent component in the degradation machinery, the anaphase-promoting complex/cyclosome in complex with CDH1 (APC/C^CDH1^) has been shown to ubiquitylate TACC3 proteins during late mitosis.^30^ To examine whether APC/C^CDH1^ participates in the SNIPER(TACC3)-dependent degradation of the TACC3 protein, we downregulated key components of APC/C^CDH1^ by the siRNAs. Knockdown of CDH1, a substrate-recognition subunit for TACC3, completely abolished the reduction of TACC3 protein by SNIPER(TACC3)-1 treatment, while the knockdown of CDC20, another substrate-recognition subunit in APC/C for different proteins,^31^ scarcely attenuated the TACC3 reduction (Figure 3b). In addition, knockdown of APC11, a RING H2 subunit recruiting E2 enzyme,^31^ and APC3, a core component of APC/C, also abrogated the TACC3 reduction by SNIPER(TACC3). We repeated the experiments with three different siRNAs against CDH1, and the abrogation of TACC3 protein knockdown was confirmed (Figure 3c). These results indicate that APC/C^CDH1^ is required for the SNIPER(TACC3)-induced degradation of the TACC3 protein.

**Figure 4:**
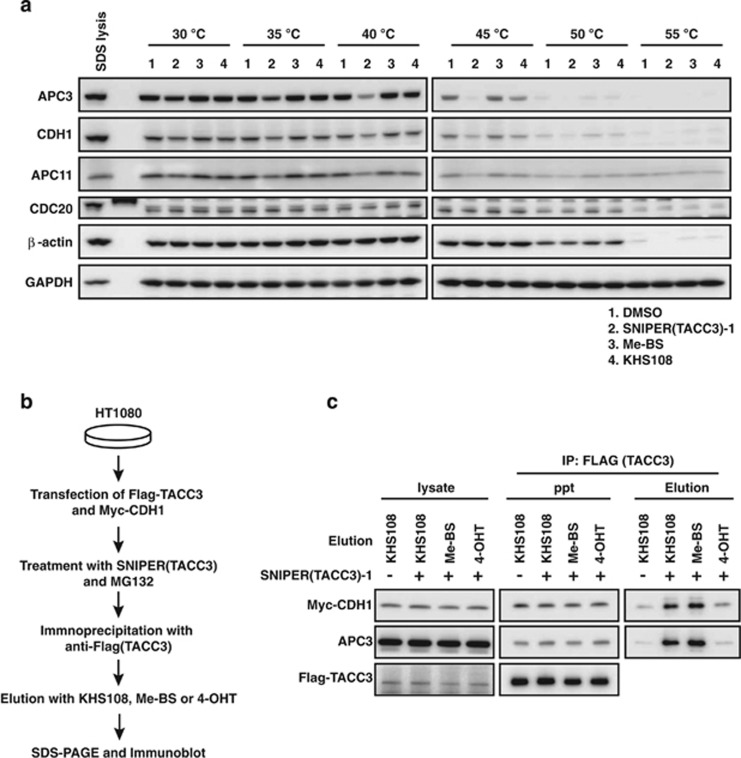
SNIPER(TACC3) physically interacts with APC/C^CDH1^ and increases the TACC3–APC/C^CDH1^ interaction. (**a**) Cell lysates were mixed with 100 *μ*M of SNIPER(TACC3)-1, Me-BS or KHS108 and analyzed by a thermal shift assay. (**b**) Scheme of the experimental procedure to detect the SNIPER(TACC3)-mediated interaction of TACC3 and APC/C^CDH1^. (**c**) HT1080 cells were co-transfected with the expression vector of Flag-TACC3 and Myc-CDH1. After 40 h, cells were treated with 25 *μ*M of MG132 in the presence or absence of 30 *μ*M of SNIPER(TACC3)-1 for 3 h. Cells were lysed in immunoprecipitated (IP) lysis buffer and Flag-TACC3 was IP with an anti-FLAG antibody. The precipitates were eluted with 2 mM of KHS108, Me-BS or 4-OHT. Total cell lysates, immunoprecipitates after elution and eluted fractions were western blotted with the indicated antibodies. DMSO, dimethyl sulfoxide; Elution, eluted fraction; lysate, total lysate; ppt, immunoprecipitate after elution; SDS-PAGE, sodium dodecyl sulfate-polyacrylamide gel electrophoresis

To confirm the physical interaction of SNIPER(TACC3) and APC/C^CDH1^, we performed a thermal shift assay that is based on the biophysical principle of ligand-induced change of thermal sensitivity of target proteins^32^ (Figure 4a). Multiple aliquots of the cell lysates were mixed with each compounds (SNIPER(TACC3)-1, Me-BS or KHS108) and heated to graded temperatures. After cooling, the lysates were centrifuged to precipitate unfolded proteins, and the supernatants were analyzed by western blotting. The thermal sensitivity of APC/C^CDH1^ components (APC3, APC11 and CDH1) were changed by addition of SNIPER(TACC3)-1, but not by Me-BS and KHS108. On the other hand, the thermal sensitivity of CDC20 was not affected by these compounds. These results indicate the physical interaction of SNIPER(TACC3) and APC/C^CDH1^.

Next we examined whether SNIPER(TACC3) treatment increases the interaction between TACC3 and APC/C^CDH1^ by crosslinking these proteins. As TACC3 inherently interacts with APC/C^CDH1^, we tried to discriminate the SNIPER(TACC3)-mediated interaction of these proteins by the following procedure (Figure 4b). HT1080 cells were transiently co-transfected with the expression vectors of Flag-TACC3 and Myc-CDH1 and then treated with MG132 in the presence or absence of SNIPER(TACC3)-1. The cell lysates were immunoprecipitated with anti-FLAG (TACC3) antibody, and the immunoprecipitates were eluted with an excess amount of either KHS108 or Me-BS to detect the APC/C^CDH1^ components that had associated with TACC3 depending on SNIPER(TACC3) (Supplementary Figure S1). Figure 4c shows a significant increase of Myc-CDH1 and endogenous APC3 in the fractions eluted with KHS108 and Me-BS but not with 4-hydroxy tamoxifen (4-OHT) as a control compound. Me-BS eluted the proteins probably by disturbing the binding of SNIPER(TACC3) to APC/C^CDH1^. These results strongly suggest that there are two types of interaction between TACC3 and APC/C^CDH1^: one is an inherent interaction independent of SNIPER(TACC3) (Supplementary Figure S1A), and the other is a SNIPER(TACC3)-mediated interaction (Supplementary Figure S1B). SNIPER(TACC3) is likely to increase the amount of TACC3 associated with APC/C^CDH1^ via the second mechanism.

**Figure 5:**
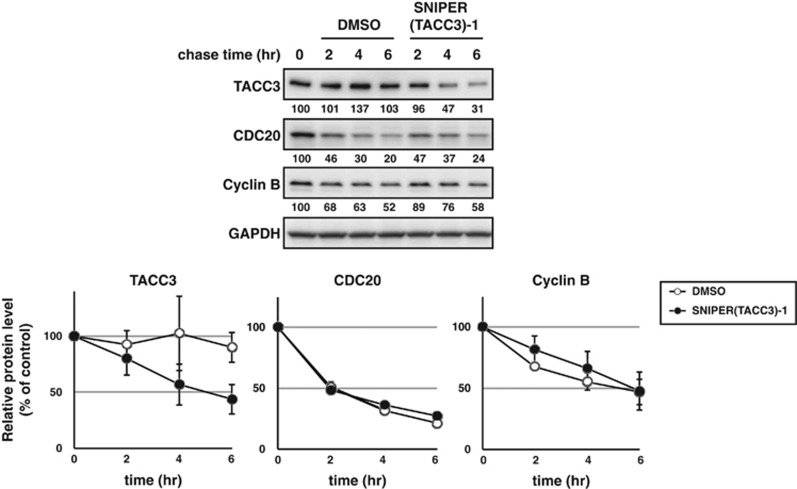
Turnover rate of APC/C substrate proteins. HT1080 cells were treated with 10 *μ*g/ml of cycloheximide in the presence or absence of 30 *μ*M of SNIPER(TACC3)-1 for the indicated periods. Whole-cell lysates were analyzed using western blotting with the indicated antibodies (top). The expression levels of TACC3, CDC20 and Cyclin B were normalized by glyceraldehyde 3-phosphate dehydrogenase (GAPDH), and the relative levels compared with time 0 were evaluated. The graphs are the means±S.D. of a representative experiment performed in triplicate (bottom). DMSO, dimethyl sulfoxide

As the SNIPER(TACC3) interacts with APC/C^CDH1^, it is possible that it directly activates APC/C^CDH1^ and promotes the degradation of many target proteins. Therefore, we explored the possibility that SNIPER(TACC3) may facilitate the degradation of a variety of proteins ubiquitylated by APC/C^CDH1^. As cyclin B and CDC20 are known to be ubiquitylated by APC/C^CDH1^,^33^ the turnover of these proteins was examined after SNIPER(TACC3) treatment. The result showed that TACC3 was scarcely degraded until 6 h in control cells, but rapidly degraded in the SNIPER(TACC3)-treated cells, with a half-life of approximately 5 h (Figure 5). However, the degradation of cyclin B and CDC20 was unaffected by SNIPER(TACC3)-1 treatment. This result suggests that SNIPER(TACC3) specifically facilitates degradation of the TACC3 protein. We also investigated cell cycle distribution of HT1080 cells treated with SNIPER(TACC3)-1 for 4 h (Supplementary Figure S2). The result showed that SNIPER(TACC3) did not affect the cell cycle distribution, suggesting that the degradation of TACC3 by SNIPER(TACC3)-1 is not due to arresting cells in a certain cell cycle phase where the protein is preferentially degraded.

### Selective cytotoxicity of SNIPER(TACC3) against cancer cells

**Figure 6:**
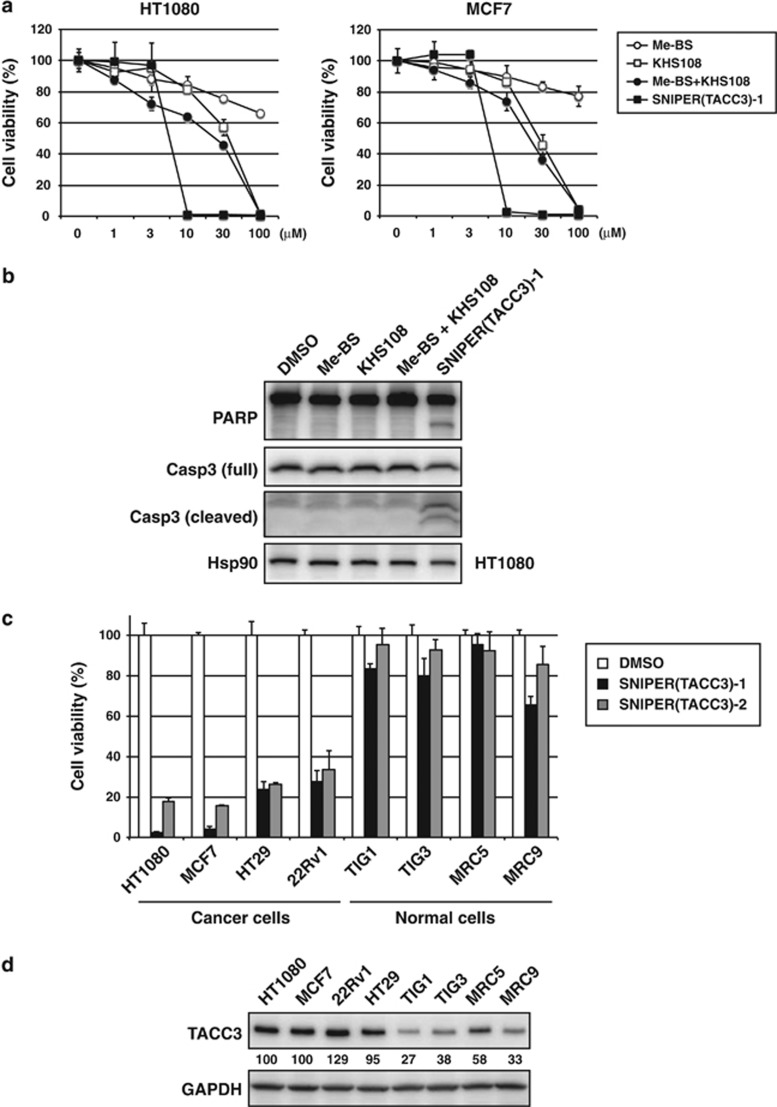
SNIPER(TACC3) selectively induces cancer cell death expressing a large amount of the TACC3 protein. (**a** and **c**) Cells were treated with the indicated compounds for 48 h, and cell viability was measured by WST-8 cell proliferation assay. The graphs show the means±S.D. of a representative experiment performed in triplicate. (**b**) Cells were treated with 10 *μ*M of the indicated compounds for 24 h, and the whole-cell lysates were analyzed by western blotting with the indicated antibodies. (**d**) Whole-cell lysates from exponentially growing cells were analyzed by western blotting. The numbers below the TACC3 panel show the relative level of TACC3 compared with HT1080 cells normalized by glyceraldehyde 3-phosphate dehydrogenase (GAPDH). DMSO, dimethyl sulfoxide

In some cancer cell lines, depletion of TACC3 induces postmitotic cell cycle arrest or mitotic cell death.^15, 16, 17, 18, 19^ TACC3 deletion was also shown to result in a massive apoptotic regression of mouse thymic lymphoma *in vivo* without any overt abnormalities in normal tissues.^21^ Therefore, we tested the effect of SNIPER(TACC3) on the cell viability of cancer cells. HT1080 and MCF7 cells were treated with Me-BS, KHS108, their combination or SNIPER(TACC3)-1 for 48 h, and cell viability was determined. SNIPER(TACC3) at ≥10 *μ*M efficiently killed the cancer cells (Figure 6a), which is consistent with the protein knockdown activity under long-term (24 h) treatment (Figure 1b). On the other hand, individual or combined treatment with Me-BS and KHS108 exhibited mild effects on the cell viability up to 30 *μ*M. In line with this, SNIPER(TACC3)-1 at 10 *μ*M, but not Me-BS, KHS108 or their combination, induced caspase-3 activation and PARP cleavage in the cells, suggesting SNIPER(TACC3) induces apoptotic cell death (Figure 6b). We also examined the effects of SNIPER(TACC3) on the viability of other cancer cell lines and normal cells. Figure 6c shows that the cell viability was greatly reduced by SNIPER(TACC3)-1 and -2 in human cancer cells but minimally in normal human fibroblasts. Selective induction of apoptosis in cancer cells by SNIPER(TACC3) was confirmed by Annexin V/propidium iodide (PI) staining (Supplementary Figure S3) and flow cytometric analysis (Supplementary Figure S4). To understand the mechanism behind the selective toxicity of SNIPER(TACC3) against cancer cells, we compared the level of TACC3 protein. Figure 6d shows that the expression level of TACC3 protein in cancer cells was much higher than that in normal cells. Depletion of TACC3 by siRNA showed only mild effect on cell cycle distribution and cell viability in these cell lines (Supplementary Figure S5). However, when SNIPER(TACC3)-induced degradation of TACC3 protein was abrogated by siRNA against APC/C^CDH1^ components (Figures 3b and c), SNIPER(TACC3)-induced cancer cell death was seriously suppressed (Supplementary Figure S6). These results suggest that SNIPER(TACC3) selectively kills cancer cells expressing a large amount of the TACC3 protein, and the degradation of TACC3 protein has an important role in the SNIPER(TACC3)-induced cancer cell death.

## Discussion

Protein knockdown with SNIPER technology selectively degrades target proteins by small molecules composed of two ligands, one against cIAP1 and the other against a target protein. Theoretically, this method enables the induction of a rapid degradation of a target even if it is a long-lived protein, which stands in contrast with the repression of protein synthesis by siRNA and antisense oligonucleotides that require a longer time to achieve efficient knockdown. We have developed a series of SNIPER compounds targeting a variety of proteins, including CRABP-II and ER*α*.^22, 23, 24, 25, 26, 27, 28^ These SNIPERs are designed to induce cIAP1-mediated ubiquitylation and proteasomal degradation of the target proteins, and they actually reduce the target proteins by the expected mechanism.

In this study, on the basis of our previous success, we designed and synthesized SNIPER(TACC3) to target TACC3 protein for degradation. The SNIPER(TACC3) induces proteasomal degradation of the TACC3 protein in the cells, as intended. Unexpectedly, however, cIAP1 is not involved in the SNIPER(TACC3)-mediated protein knockdown of the TACC3 protein. Instead, APC/C^CDH1^ has an important role in the degradation of the TACC3 induced by SINPER(TACC3). As APC/C^CDH1^ is a physiological E3 ligase for TACC3^30^ and SNIPER(TACC3) interacts with APC/C^CDH1^, it is possible that SNIPER(TACC3) non-specifically activates APC/C^CDH1^ in order to facilitate the ubiquitylation and degradation of many different proteins. However, this is not the case, because degradation of cyclin B and CDC20, both of which are substrates for APC/C^CDH1^-mediated ubiquitylation as well, were not facilitated by SNIPER(TACC3). A thermal shift assay and co-immunoprecipitation followed by elution with individual ligands (KHS108 and Me-BS) (Figure 4) suggest that SNIPER(TACC3) crosslinks TACC3 and APC/C^CDH1^, thereby increasing the interaction of these proteins (Supplementary Figure S1). Thus, unlike other SNIPERs that induce cIAP1-mediated ubiquitylation of target proteins, SNIPER(TACC3) induces APC/C^CDH1^-mediated ubiquitylation of the TACC3 protein. In all cases, the ubiquitylated proteins were subjected to proteasomal degradation, resulting in a reduction of the target.

TACC3 has a pivotal role on the regulation of spindle formation. When the TACC3 protein is depleted by siRNA or genetic ablation, cell lines show mitotic or postmitotic arrest and occasionally undergo apoptosis.^7, 15, 16, 19, 20^ However, as thus far examined with SNIPER(TACC3)s, we did not observe any mitotic arrest but rather cell death via apoptosis. This may be due to an insufficient reduction of the TACC3 protein and/or simultaneous reduction of anti-apoptotic cIAP1 protein by SNIPER(TACC3) treatment. It is also possible that SNIPER(TACC3) additionally affect a cellular function related to cell death. In the case of SNIPER(ER), there is a robust production of reactive oxygen species (ROS) after ER*α* degradation that results in necrotic cell death accompanied by the release of HMGB1 from the cells.^28^ SNIPER(TACC3), however, does not induce a robust ROS production in cells.

One of the interesting feature of SNIPER(TACC3) is the ability to induce apoptosis selectively in cancer cells expressing large amounts of TACC3 protein. As TACC3 level is higher in actively dividing cells, SNIPER(TACC3) might selectively kill cancer cells that are more actively proliferating than non-tumor cells. Degradation of TACC3 seems to have an important role in the SNIPER(TACC3)-induced apoptosis, because downregulation of APC/C^CDH1^-components by siRNA abrogates the SNIPER(TACC3)-induced TACC3 degradation (Figure 3), and suppresses cell death (Supplementary Figure S6), though TACC3 depletion by siRNA is not enough to induce cell death in these cancer cells (Supplementary Figure S5),

Recently, TACC3 has attracted increasing attention as a target for cancer therapy,^21, 34, 35, 36, 37, 38, 39, 40, 41^ and inhibitors of TACC3 have been reported to possess anti-tumor activity.^42^ As SNIPER(TACC3) exhibits selective toxicity to cancer cells aberrantly expressing large amount of the TACC3 protein as compared with normal cells, protein knockdown is a strategy for disrupting TACC3 function in cancer cells.

## Materials and Methods

### Design and synthesis of SNIPER(TACC3)-1 and -2

The small-molecule KHS101 and its derivative KHS108 have been reported to interact with the TACC3 protein.^43^ Accordingly, KHS101 and bestatin were used as TACC3 and cIAP1 ligands, respectively. We designed the hybrid molecules SNIPER(TACC3)-1 and -2 in which KHS108 is linked to bestatin via a linker having a different polyethylene glycol (PEG) unit (Figure 1a). The attachment point of KHS108 to the PEG linker was determined at the end of the methoxyethylaminocarbonyl group, which does not affect the neuronal differentiation activities of KHS101 derivatives according to the literature.^43^

The chemical synthesis and physicochemical data on SNIPER(TACC3)-1 and -2 are provided in the Supplementary Information.

### Plasmids

The cDNA encoding human TACC3 was amplified by PCR from HepG2 cDNA and cloned into a pCMV5-FLAG expression vector. The correct cDNA sequence was confirmed. pcDNA3-Myc-CDH1 was described previously.^44^

### Cell culture and transfection

Human fibrosarcoma HT1080, human osteosarcoma U2OS, human colon adenocarcinoma HT29 and human fetal lung fibroblasts TIG1, TIG3, MRC5 and MRC9 were maintained in Dulbecco's modified Eagle's medium supplemented with 10% fetal bovine serum (FBS) and 100 *μ*g/ml of kanamycin. In some experiments, MCF7 cells were precultured in the media containing estrogen-depleted serum as previously described.^28^ Human breast adenocarcinoma MCF7 and human prostate carcinoma 22Rv1 were maintained in RPMI 1640 medium containing 10% FBS and 100 *μ*g/ml of kanamycin. HT1080 cells were transiently transfected with gene-specific short interfering RNA (siRNA) or negative control siRNA (QIAGEN, Valencia, CA, USA) using lipofectamine RNAi MAX reagent (Invitrogen, Tokyo, Japan). The siRNA sequences used in this study were: human cIAP1-1 (5′-UCUAGAGCAGUUGAAGACAUCUCUU-3'); cIAP1-2 (5′-GCUGUAGCUUUAUUCAGAAUCUGGU-3′); cIAP1-3 (5′-GGAAAUGCUGCGGCCAACAUCUUCA-3'); CDH1-1 (5′-GGAUUAACGAGAAUGAGAA-3'); CDH1-2 (5′-UGAGAAGUCUCCCAGUCAG-3'); CDH1-3 (5′-GCACGGAGACCGCUUCAUC-3'); CDC20 (5′-GUCCCCCCGGAAACCCACC-3'); APC11-1 (5′-GGUGAAGAUUAAGUGCUGG-3'); APC11-2 (5′-CGAUGAGAACUGUGGCAUC-3'); and APC3 (5′-GGAAAUAGCCGAGAGGUAA-3').

### Western blotting

Cells were lysed with SDS lysis buffer (0.1 M Tris-HCl at pH 8.0, 10% glycerol, 1% SDS) and boiled for 10 min. The protein concentration was measured by the BCA method (Pierce, Rockford, IL, USA), and the lysates containing an equal amount of protein were separated by SDS-PAGE, transferred to PVDF membranes (Millipore, Darmstadt, Germany) for western blotting using the appropriate antibodies. The immunoreactive proteins were visualized using the Immobilon Western chemiluminescent HRP substrate (Millipore), and light emission was quantified with a LAS-3000 lumino-image analyzer (Fuji, Tokyo, Japan). The antibodies used in this study were: anti-TACC3 rabbit monoclonal antibody (mAb) (Cell Signaling Technology, Danvers, MA, USA; 8069), anti-TACC3 rabbit polyclonal antibody (pAb) (Santa Cruz, Dallas, TX, USA; sc-22773), anti-cIAP1 goat pAb (R&D systems, Minneapolis, MN, USA; AF8181), anti-GAPDH pAb (Santa Cruz, sc-25778 HRP), anti-CDH1 mouse mAb (Calbiochem, La Jolla, CA, USA; Ab-2), anti-CDC20 rabbit pAb (Santa Cruz, sc-8358), anti-APC11 rabbit pAb (Acris, San Diego, CA, USA; R1503), anti-APC3 mouse mAb (Santa Cruz, sc-13154), anti-cyclin B (Santa Cruz, sc-245), anti-PARP rabbit mAb (Cell Signaling Technology, 9532), anti-caspase-3 rabbit pAb (Santa Cruz, sc-7148) and anti-Hsp90 mouse mAb (BD Transduction, San Jose, CA, USA; 610419), anti-CRABP-II rabbit pAb (Abcam, Cambridge, UK; ab72099), anti-ER*α* rabbit mAb (Cell Signaling Technology, 8644), and anti-*β*-actin mouse mAb (Sigma, St. Louis, MO, USA; A2228).

### Ubiquitylation assay

HT1080 cells were transfected with pCMV5-FLAG-TACC3 and pcDNA3-HA-ubiquitin for 40 h The cells were then incubated with the indicated compounds in the presence of MG132 (25 *μ*M) for 3 h before being harvested and lysed in SDS lysis buffer. The cell lysates were boiled for 10 min, diluted 10 times with IP lysis buffer (10 mM Hepes at pH7.4, 142.5 mM KCl, 5 mM MgCl_2_, 1 mM EGTA and 1% NP-40) and immunoprecipitated with anti-FLAG agarose-conjugated beads. The precipitates were extensively washed and analyzed by western blotting using an HRP-conjugated anti-HA antibody (Roche, Basel, Switzerland) or anti-ubiquitin, Lys48-Specific (Millipore, 05-1307).

### SNIPER-mediated interaction of TACC3 and APC/C^CDH1^

Cells were co-transfected for 40 h with the expression vectors of Flag-TACC3 and Myc-CDH1 and treated for 3 h with MG132 in the presence or absence of SNIPER(TACC3)-1. Cells were lysed with IP lysis buffer (10 mM Hepes at pH 7.4, 142.5 mM KCl, 5 mM MgCl_2_, 1 mM EGTA and 0.1% Triton X-100) containing a protease inhibitor cocktail, rotated for 15 min at 4 °C and centrifuged at 15 000 r.p.m. for 10 min at 4 °C to obtain the supernatants. The lysates, which had been precleared with naked protein G-sepharose, were immunoprecipitated with anti-FLAG agarose-conjugated beads for 2 h at 4 °C. The precipitates were washed with IP lysis buffer four times and eluted by mild vortexing with IP lysis buffer containing the compounds for 15 min at room temperature. After centrifugation at 15 000 r.p.m. for 1 min, the eluted fractions (the supernatants) were obtained and analyzed by western blotting.

### Thermal shift assay

Thermal shift assay was performed as previously described.^32^ Cells were harvested and washed with PBS. The cells were suspended in kinase buffer (25 mM Tris(hydroxymethyl)-aminomethane hydrochloride (Tris-HCl, pH 7.5), 5 mM beta-glycerophosphate, 2 mM dithiothreitol, 0.1 mM sodium vanadium oxide, 10 mM magnesium chloride) (Cell Signaling Technology) supplemented with protease inhibitor cocktail. The cell suspensions were freeze-thawed three times using liquid nitrogen and centrifuged at 15 000 r.p.m. for 10 min at 4 °C to obtain the supernatants (lysate). The cell lysates were divided into four aliquots, with each aliquot being treated with each compound or DMSO (control). After 10–30 min incubation at room temperature, the respective lysates were divided into smaller (50 *μ*l) aliquots and heated individually at graded temperatures for 3 min (PCR thermal cycler, Applied Biosystems/Life Technologies, Carlsbad, CA, USA) followed by cooling for 3 min at room temperature. The heated lysates were centrifuged at 15 000 r.p.m. for 10 min at 4 °C in order to separate the soluble fractions from precipitates. The supernatants were transferred to new microtubes and analyzed by SDS-PAGE followed by western blotting.

### Cell viability assay

Cell viability was determined using water-soluble tetrazolium WST-8 (4-[3-(2-methoxy-4-nitrophenyl)-2-(4-nitrophenyl)-2H-5-tetrazolio]-1,3-benzene disulfonate) for the spectrophotometric assay according to the manufacturer's instructions (Dojindo, Tokyo, Japan). Cells were seeded at a concentration of 5 × 10^3^ cells per well in a 96-well culture plate. After 24 h, cells were treated with the indicated compounds for 48 h. The WST-8 reagent was added, and the cells were incubated for 0.5 h at 37 °C in a humidified atmosphere of 5% CO_2_. The absorbance at 450 nm of the medium was measured using an EnVision Multilabel Plate Reader (PerkinElmer, Waltham, MA, USA).

### Measurement of apoptosis by flow cytometer

Apoptosis was analyzed with an Annexin V-FITC Apoptosis Detection Kit (BioVision, Milpitas, CA, USA). After treatment, cells were gently trypsinized and washed with serum-containing medium. Cells were collected by centrifugation, and additionally washed with PBS, and resuspended in Binding Buffer. The cells were stained with annexin V-FITC and PI at room temperature for 5 min in the dark, according to the manufacturer's instructions, and analyzed on a FACScan flow cytometer (Becton Dickinson, Braintree, MA, USA).

### Cell cycle analysis

After treatment, cells were gently trypsinized and washed with serum-containing medium. Cells were collected by centrifugation, and additionally washed with PBS, and fixed in 70% ice-cold ethanol for 1 h on ice. The cells were then washed, treated with 1 mg/ml RNase A for 1 h at 37 °C and stained in PI solution (50 *μ*g/ml in 0.1% sodium citrate, 0.1% NP-40). The stained cells were analyzed on a FACScan flow cytometer (Becton Dickinson).

## Abbreviations

TACC3: transforming acidic coiled-coil-3
UPS: ubiquitin–proteasome system
SNIPER: specific and non-genetic IAP-dependent protein eraser
cIAP1: cellular inhibitor of apoptosis protein 1
CRABP-II: cellular retinoic acid binding protein-II
ER*α*: estrogen receptor *α*
Me-BS: methyl-bestatin
PEG: polyethylene glycol
siRNA: small interfering RNA
APC/C: anaphase-promoting complex/cyclosome
4-OHT: 4-hydroxy tamoxifen
ROS: reactive oxygen species
PI: propidium iodide

## Notes

K Nagai and N Cho are employees of Takeda Pharmaceutical Co., Ltd. (Osaka, Japan). M Naito received a research fund from Takeda Pharmaceutical Co., Ltd. The other authors declare no conflict of interest.
