# Supplementary material for: Cancer cell death induced by novel small molecules degrading the TACC3 protein via the ubiquitin–proteasome pathway
Source: Cell Death Dis. 2014 Nov 6;5(11):e1513–. doi: 10.1038/cddis.2014.471 (PMC4260729; doi:10.1038/cddis.2014.471)
Supplement: Supplementary Information [file cddis2014471x1.doc]

**Supplementary information**

Chemical synthesis of SNIPER(TACC3)-1, 2 was performed as illustrated in Scheme 1. Starting from the commercially available ethyl 2-aminothiazole-4-carboxylate (**2**), the key intermediate **10** was synthesized using a modified procedure as described in the literature^42^. The aminomethylthiazole **6** was prepared from **2** via a four-step protocol, i.e. Sandmeyer reaction, reduction of the ethyl ester, Suzuki coupling reaction and amination of the hydroxymethyl group. Replacement of the 4-chloro atom of **7** with isobutylamine and subsequent replacement of the 2-chloro atom with the amine **6** afforded the ester **9**, which was subjected to alkaline hydrolysis to give the carboxylic acid **10**. The amino group of the bestatin (**11**) purchased from OChem Inc. was protected by a 9-fluorenylmethyloxycarbonyl (Fmoc) group to yield compound **12**, which was then condensed with mono-Boc diamines having different PEG units (n = 1, 2) to provide the amides **13a**, **b**. After removal of the Boc group of **13a**, **b**, condensation with the carboxylic acid **10** gave the corresponding precursors **14a, b**. Final deprotection of the Fmoc group of **14a, b** with dimethylamine afforded SNIPER(TACC3)-1 (**1a**) and SNIPER(TACC3)-2 (**1b**).

**Scheme 1.** Synthesis of SNIPER(TACC3)-1, 2*^a^*

*^a^*Reagents and conditions: (a) CuSO_4_, NaBr, NaNO_2_, H_2_SO_4_, water, 0 °C, 50%; (b) LiBH_4_, THF, MeOH, 0 °C, 84%; (c) (4-(ethoxycarbonyl)phenyl)boronic acid, Pd(PPh_3_)_4_, DME–MeOH, 80 °C, 59%; (d) diphenylphosphoryl azide (DPPA), 1,8-diazabicyclo[5.4.0]undec-7-ene (DBU), THF, rt then PPh_3_, water, rt, 79%; (e) isobutylamine, EtOH, 80 °C, 77%; (f) compound **6**, *N*,*N*-diisopropylethylamine (DIEA), *n*-BuOH, reflux, 67%; (g) 1 M NaOH, MeOH, rt, 99%; (h) 9-fluorenylmethyloxycarbonyl chloride (Fmoc-Cl), K_2_CO_3_, THF–water, rt, 81%; (i) *tert*-butyl (2-(2-(2-aminoethoxy)ethoxy)ethyl)carbamate [for **14a**], *tert*-butyl (2-(2-(2-(2-aminoethoxy)ethoxy)ethoxy)ethyl)carbamate [for **14b**], 1-ethyl-3-(3-dimethylaminopropyl)carbodiimide (EDCI), 1-hydroxybenzotriazole hydrate (HOBt·H_2_O), THF, rt, 86–87%; (j) 4 M HCl in cyclopentyl methyl ether (CPME), THF, rt; (k) compound **10**, 1-[bis(dimethylamino)methylene]-1*H*-1,2,3-triazolo[4,5-b]pyridinium 3-oxid hexafluorophosphate (HATU), DIEA, 0 °C, 87–99%; (l) 2M dimethylamine in MeOH, rt, 84–89%.

**Physicochemical Data on SNIPER(TACC3)-1, 2**

The proton nuclear magnetic resonance (^1^H NMR) spectra were determined on a Bruker AVANCE II (300 MHz) spectrometer. Chemical shifts for ^1^H NMR were reported in parts per million (ppm) downfield from tetramethylsilane (δ) as the internal standard in deuterated solvent and the coupling constants (*J*) are in Hertz (Hz). The following abbreviations are used for spin multiplicity: s = singlet, d = doublet, dd = doublet of doublet, m = multiplet, and br s = broad singlet. Mass spectra (MS) were acquired using a Shimadzu UFLC/MS (Prominence UFLC high pressure gradient system/LCMS-2020) operating in electron spray ionization mode (ESI+). The column used was an L-column 2 ODS (3.0 x 50 mm I.D., 3 μm, CERI, Japan) with a temperature of 40 °C and a flow rate of 1.5 mL/min. Mobile phase A was 0.05% trifluoroacetic acid (TFA) in ultrapure water. Mobile phase B was 0.05% TFA in acetonitrile (MeCN) which was increased linearly from 5% to 90% over 2 minutes and 90% over the next 1.5 minutes, after which the column was equilibrated to 5% for 0.5 minutes. Analytical HPLC for purity determination was performed using a Shimadzu LC-20AD, equipped with an L-column2 ODS (3.0 x 30 mm I.D., 2 μm-particle size, CERI, Japan), elutied with 0.1% TFA in ultrapure water (Mobile phase A), and 0.1% TFA in MeCN (Mobile phase B), using the following elution gradient of 5% B to 90% B over 2.0 min followed by 90% B isocratic over 1.3 min at a flow rate of 1.2 mL/min (detection at 220 nm).

***N*-((11*S,*14*S,*15*R*)-15-Amino-14-hydroxy-11-isobutyl-10,13-dioxo-16-phenyl-3,6-dioxa-9,12-diazahexadecyl)-4-(4-(((4-(isobutylamino)pyrimidin-2-yl)amino)methyl)thiazol-2-yl) benzamide** (**SNIPER(TACC3)-1**, **1a**). ^1^H NMR (CD_3_OD) δ 0.76 – 0.99 (12H, m), 1.52 – 1.86 (4H, m), 2.64 (1H, dd, *J* = 13.3, 7.8 Hz), 2.88 (1H, dd, *J* = 13.3, 6.9 Hz), 3.08 (2H, d, *J* = 6.6 Hz), 3.25 – 3.38 (3H, m), 3.47 – 3.71 (10H, m), 3.92 (1H, d, *J* = 3.0 Hz), 4.37 – 4.47 (1H, m), 4.69 (2H, d, *J* = 0.8 Hz), 5.80 (1H, d, *J* = 6.0 Hz), 7.13 – 7.33 (6H, m), 7.60 (1H, br s), 7.88 – 8.08 (4H, m). MS *m*/*z* 804.7 (M+H)^+^. Purity 99.3% (HPLC).

***N*-((14*S,*17*S,*18*R*)-18-Amino-17-hydroxy-14-isobutyl-13,16-dioxo-19-phenyl-3,6,9-trioxa-12,15-diazanonadecyl)-4-(4-(((4-(isobutylamino)pyrimidin-2-yl)amino)methyl)thiazol-2-yl)benzamide** (**SNIPER(TACC3)-2**, **1b**). ^1^H NMR (CD_3_OD) δ 0.79 – 0.98 (12H, m), 1.53 – 1.86 (4H, m), 2.65 (1H, dd, *J* = 13.4, 7.8 Hz), 2.89 (1H, dd, *J* = 13.4, 6.9 Hz), 3.08 (2H, d, *J* = 6.3 Hz), 3.26 – 3.37 (3H, m), 3.44 – 3.72 (14H, m), 3.93 (1H, d, *J* = 3.0 Hz), 4.35 – 4.48 (1H, m), 4.69 (2H, d, *J* = 0.8 Hz), 5.80 (1H, d, *J* = 6.0 Hz), 7.11 – 7.34 (6H, m), 7.60 (1H, br s), 7.88 – 7.97 (2H, m), 7.99 – 8.08 (2H, m). MS *m*/*z* 848.8 (M+H)^+^. Purity 98.8% (HPLC).

**Supplementary Figure Legends**

Fig. S1. Schematic model of the TACC3-APC/C^CDH1^ interaction.

(A) Inherent interaction that is not affected by KHS108 or Me-BS. (B) SNIPER(TACC3)-mediated interaction that is impaired by KHS108 or Me-BS.

Fig. S2. SNIPER(TACC3) did not affect cell cycle distribution.

HT1080 cells were treated with 30 μM of SNIPER(TACC3)-1 for 4 hr and analyzed in a flow-cytometer. Cell-cycle distribution was quantified by MultiCycle software.

Fig. S3. Selective induction of cancer cell death by SNIPER(TACC3) determined by annexin V and PI staining.

Cells were treated with or without 10 μM of SNIPER(TACC3)-1 for 48 hr, stained with annexin V and PI, and analyzed in a flow-cytometer. Double negative cells (lower left) represent living cells, whereas annexin V positive (lower right), PI positive (upper left) or double positive (upper right) cells represent dead cells.

Fig. S4. Selective induction of cancer cell death by SNIPER(TACC3) determined by flow-cytometric analysis.

Cancer cells (A) and normal cells (B) were treated with or without 10 μM of SNIPER(TACC3)-1 for 48 hr. Cells were stained with propidium iodide and DNA contents were analyzed in a flow-cytometer.

Fig. S5. Depletion of TACC3 protein by siRNA did not induce cell death.

(A) Depletion of TACC3 protein by siRNA was analyzed by Western blot. (B) Cell viability was measured by WST-8 cell proliferation assay. (C, D) Cancer cells (C) and normal cells (D) were stained with PI and analyzed in a flow-cytometer. Cells were transfected with siRNA against TACC3 for 72 hr.

Fig. S6. Suppression of SNIPER(TACC3)-induced cell death by downregulation of APC/C^CDH1^.

HT1080 cells were transfected with the indicated siRNA for 24 hr and treated with 10 μM of SNIPER(TACC3)-1 for 30 hr. The cells were stained with annexin V and PI, and analyzed in a flow-cytometer.
