# Supplementary material for: Cancer cell death induced by novel small molecules degrading the TACC3 protein via the ubiquitin–proteasome pathway
Source: Cell Death Dis. 2014 Nov 6;5(11):e1513–. doi: 10.1038/cddis.2014.471 (PMC4260729; doi:10.1038/cddis.2014.471)

Supplementary Fig.S1

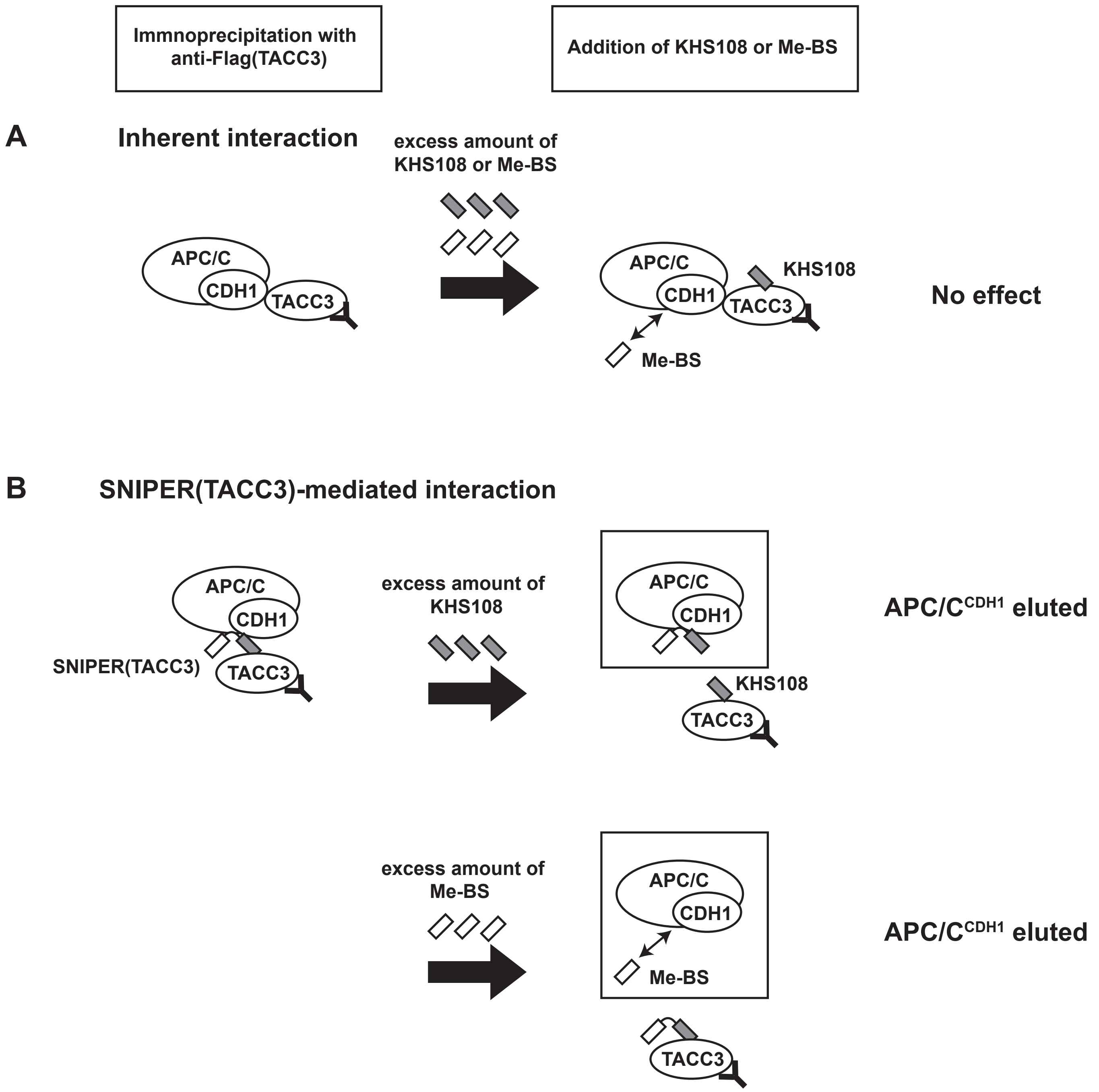

Supplementary Fig.S2

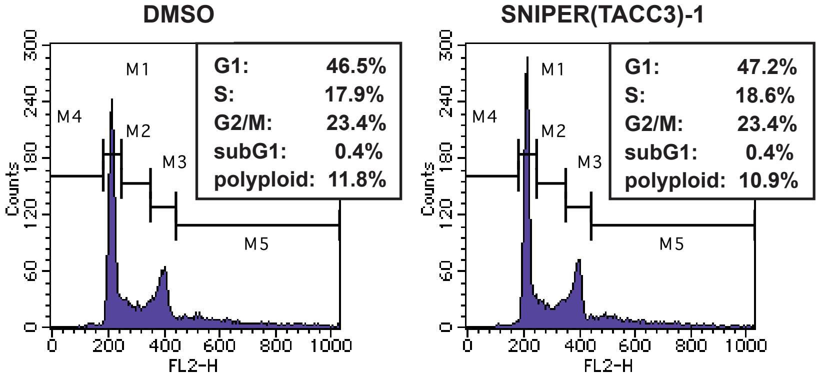

Supplementary Fig.S3

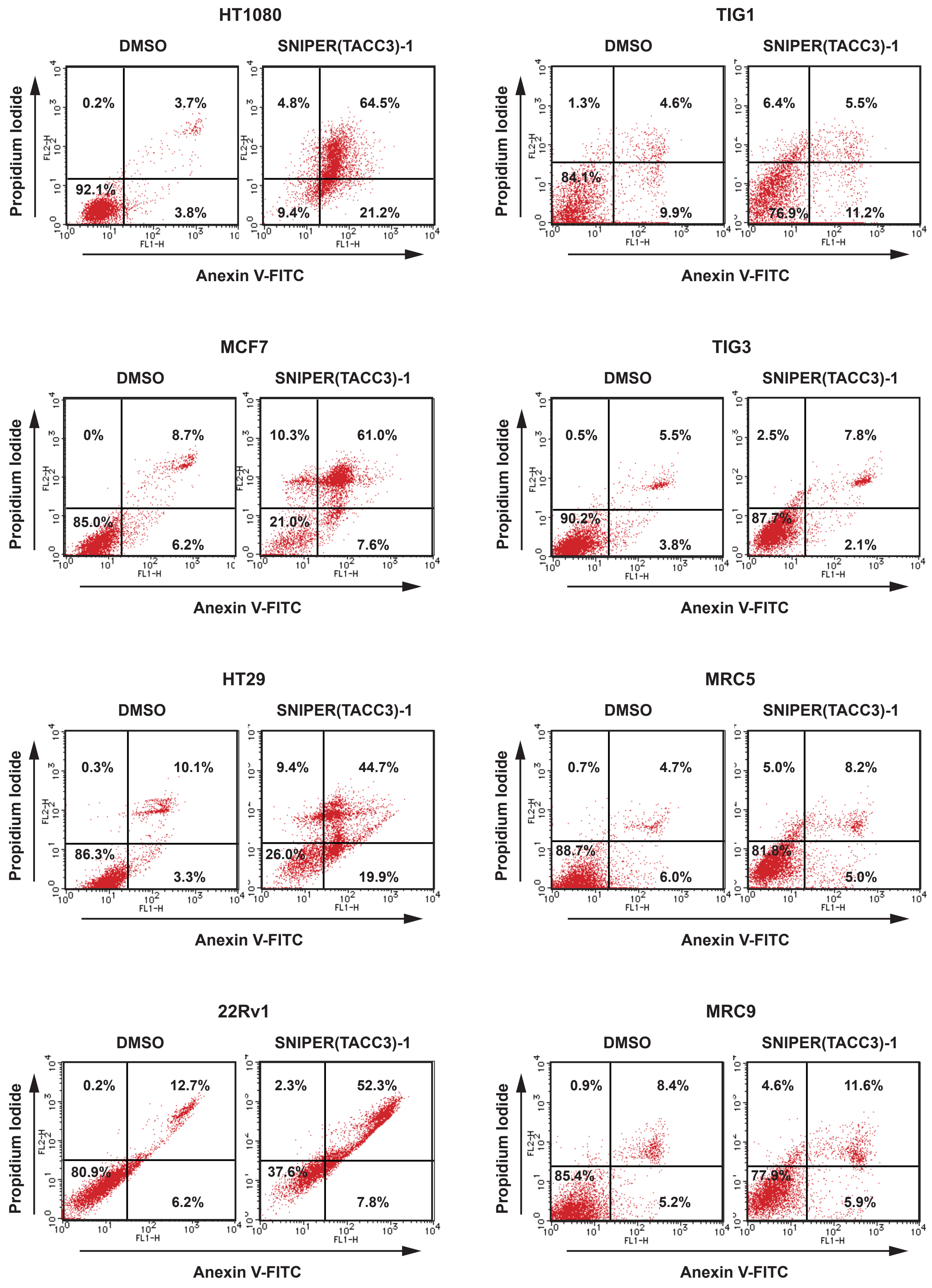

Supplementary Fig.S4

A

HT1080

MCF7

HT29

22Rv1

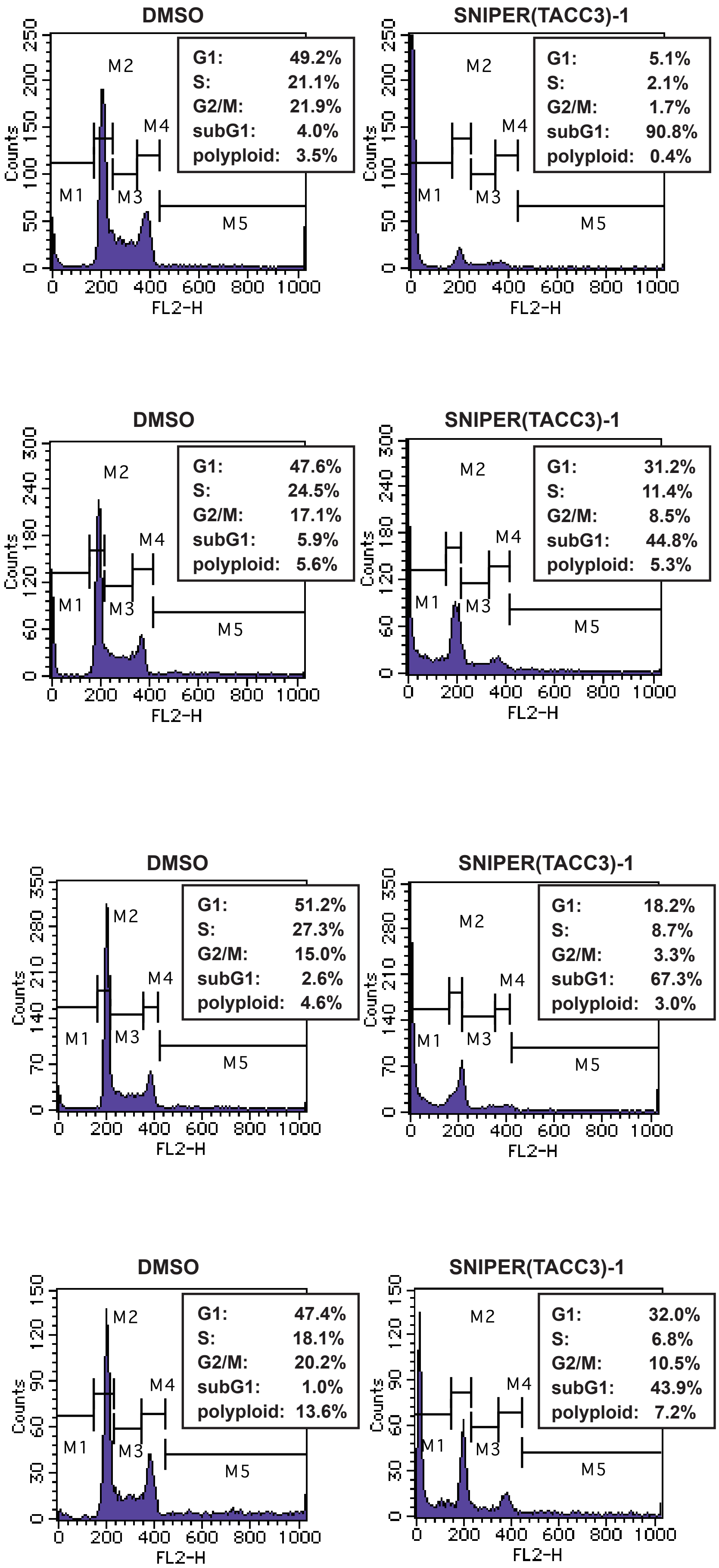

Supplementary Fig.S4

B

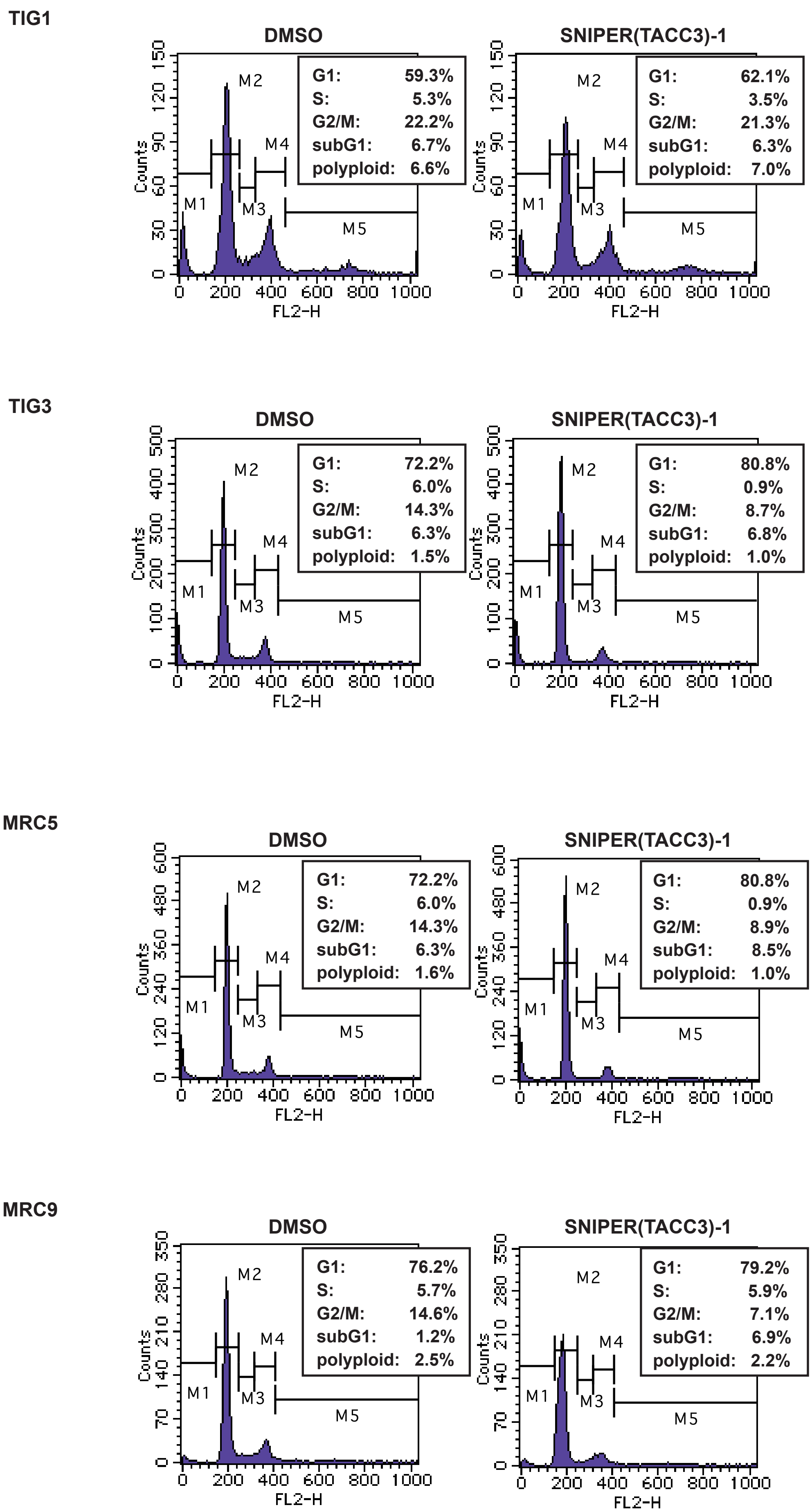

Supplementary Fig.S5

A

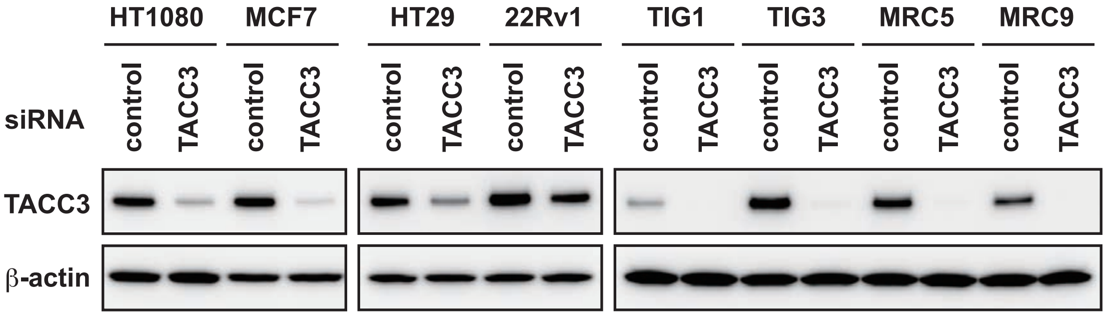

B

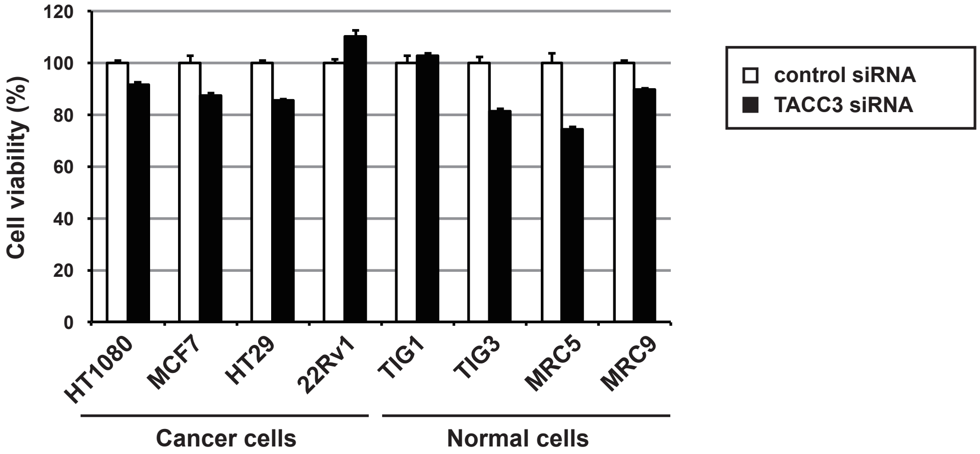

Supplementary Fig.S5

C

HT1080

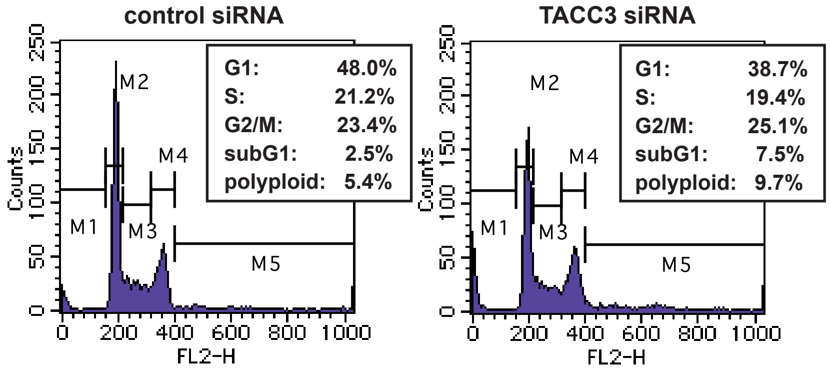

MCF7

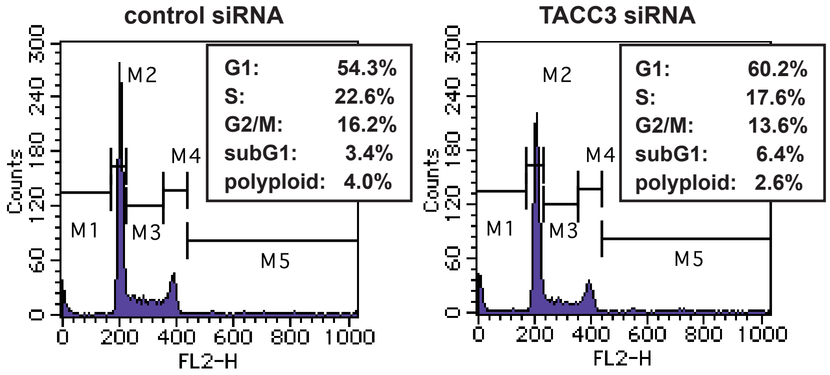

HT29

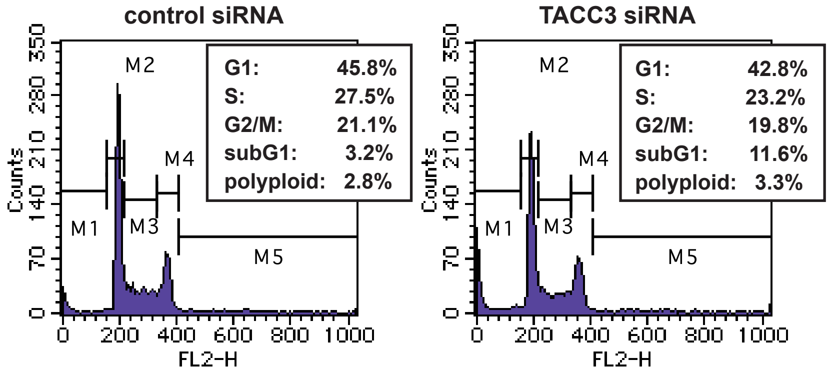

22Rv1

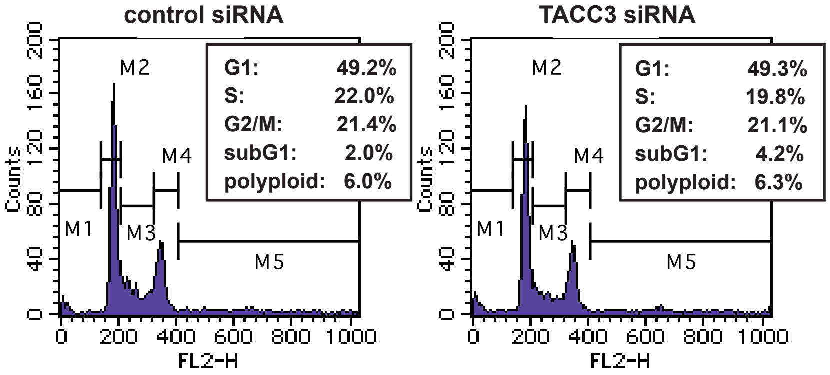

Supplementary Fig.S5

D

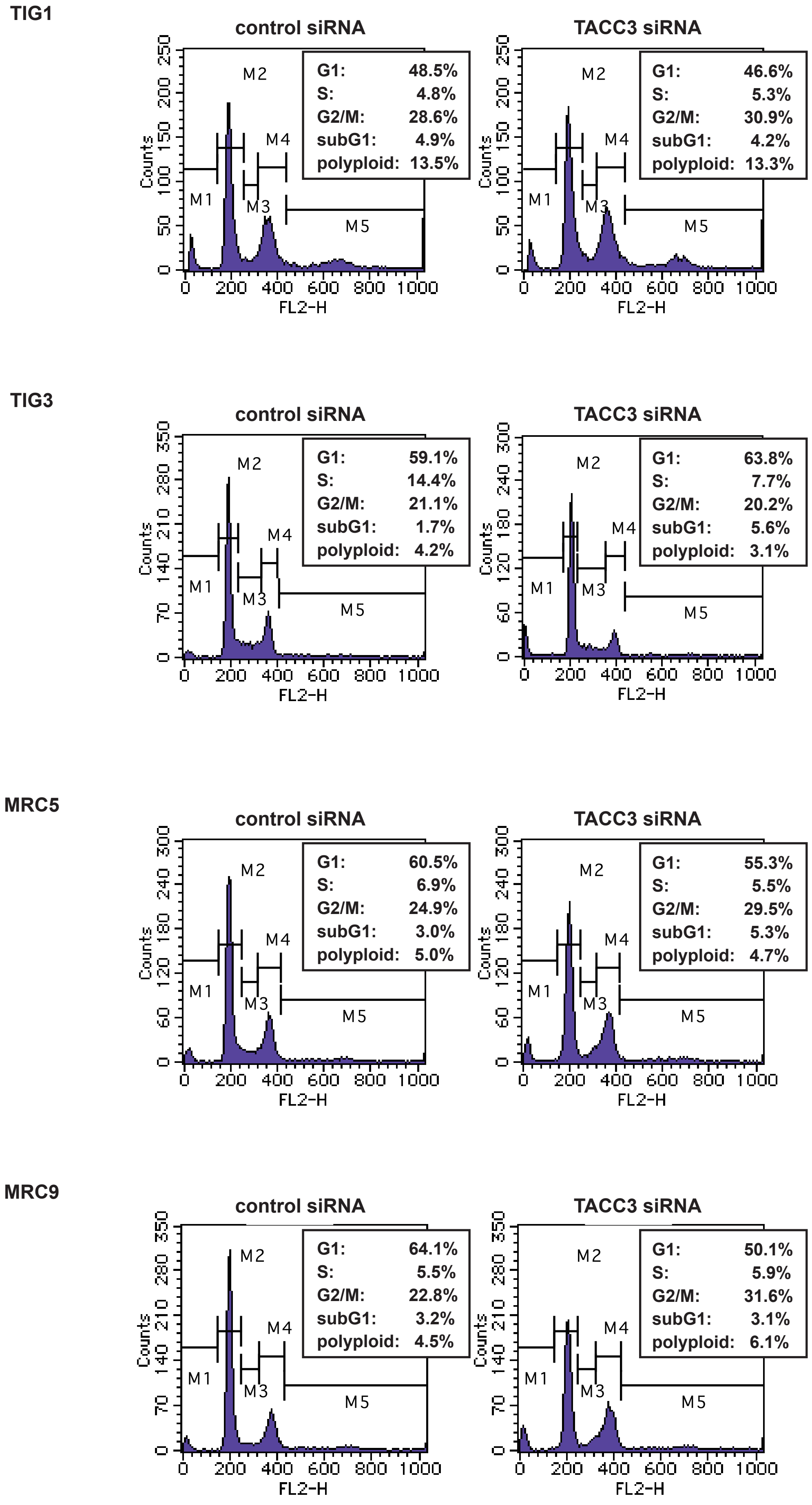

Supplementary Fig.S6

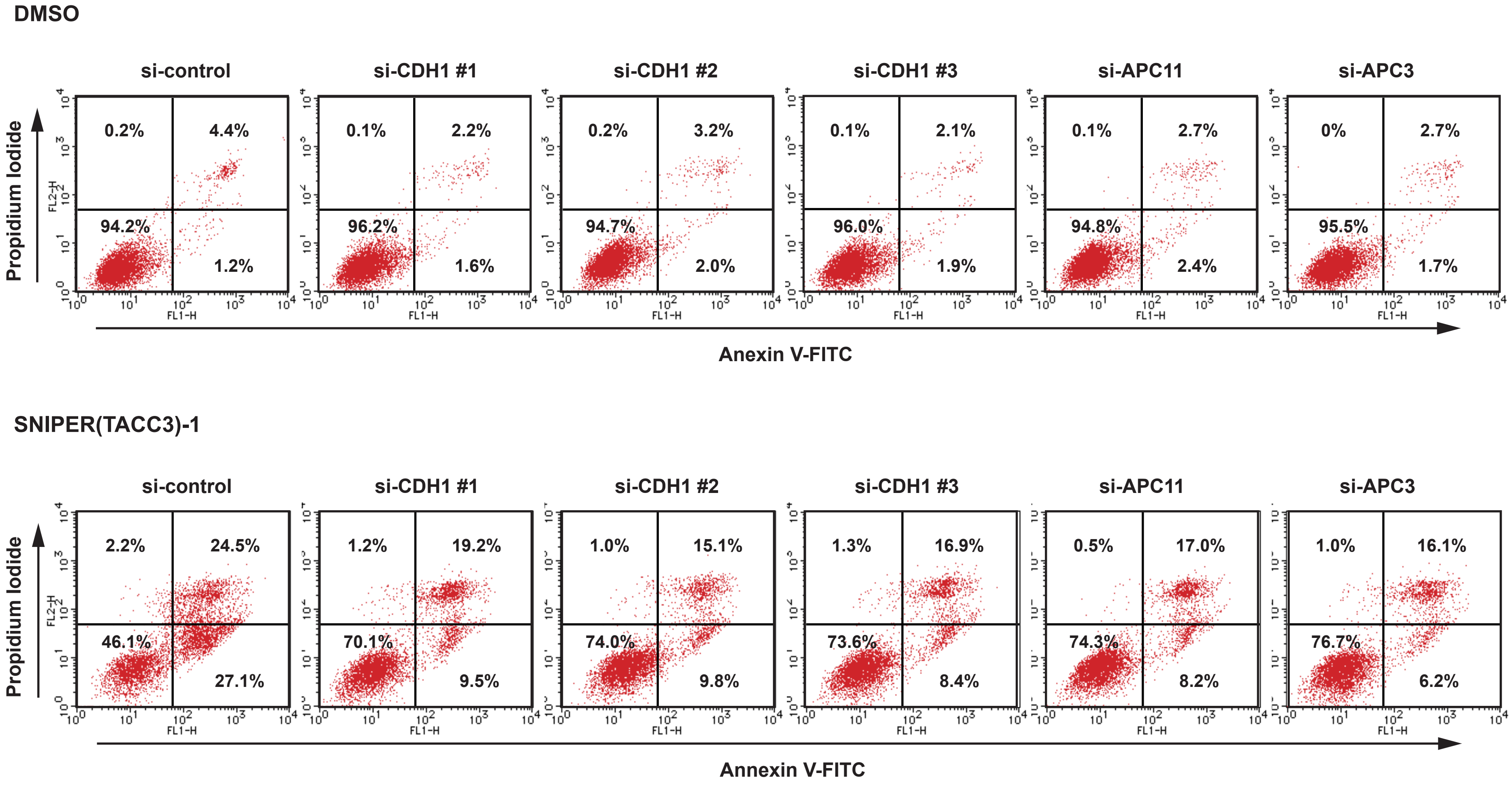

Supplement: Supplementary Figures [file cddis2014471x2.pdf]
